# *Leptospira* Seroprevalence Among Ugandan Slaughter Cattle: Comparison of Sero-Status With Renal *Leptospira* Infection

**DOI:** 10.3389/fvets.2020.00106

**Published:** 2020-02-28

**Authors:** Lordrick Alinaitwe, Clovice Kankya, Dianah Namanya, Patrick Pithua, Anou Dreyfus

**Affiliations:** ^1^College of Veterinary Medicine, Animal Resources and Biosecurity (COVAB), Makerere University, Kampala, Uganda; ^2^Virginia-Maryland College of Veterinary Medicine, Virginia Tech and University of Maryland, Blacksburg, VA, United States; ^3^Section of Epidemiology, Vetsuisse Faculty, University of Zurich, Zurich, Switzerland; ^4^Department of Medicine, Swiss Tropical and Public Health Institute, Basel, Switzerland; ^5^University of Basel, Basel, Switzerland

**Keywords:** leptospirosis, microscopic agglutination test, renal *Leptospira* infection, slaughter cattle, seroprevalence

## Abstract

Despite evidence of both human and animal *Leptospira* exposures in Uganda, the epidemiology of the disease is still not well-investigated. Contact with animals and their environments have been pointed out as potential source of infection with *Leptospira* species in humans; and cattle may be an important reservoir in Uganda. In this cross-sectional study, we estimated the prevalence of anti-*Leptospira* antibodies by the standard microscopic agglutination test (MAT); and associated risk factors among slaughtered cattle. We also compared the performance of the MAT used in this study against a *lipL32* based real time PCR (qPCR) assay previously conducted on the kidneys and urine of the same slaughter cattle as tested in this reported study. Of 500 cattle sampled, 27.8% (95% CI 23.9–32.0) tested positive (titer ≥ 100) to at least one *Leptospira* serovar, with the majority of seropositive cattle reacting to serovars Tarassovi (sg Tarassovi) (11.6%), Sejroe (Sg Sejroe) (7.8%), and Australis (Sg Australis) (5.2%). Older animals had 2.8 times (95% CI 1.0–8.2, *p*-value 0.055) greater odds of being seropositive than younger ones (<1.5 years). The sensitivity and specificity of the MAT over the qPCR were 65.9% (95% CI 50.1–79.5) and 75.9% (95% CI 71.7–79.7), respectively; with a negative predictive value of 95.8% and positive predictive value of 20.9%. In conclusion, slaughter cattle in this study were significantly exposed to pathogenic *Leptospira* species of mainly the Tarassovi, Sejroe, and Australis serogroups, with seroprevalence being higher among older cattle. The high specificity and negative predictive value of MAT as used in this study when compared to the qPCR assay may imply a rather strong association between seronegativity and absence of renal *Leptospira* infection. However, MAT predictability for renal *Leptospira* infection may be interpreted cautiously since predictive values of diagnostic tests are dependent on prevalence.

## Introduction

Leptospirosis is one of the most wide spread zoonotic bacterial diseases that is endemic in subtropical and tropical countries; accounting for a global annual incidence of 1.03 million human cases and 58,900 deaths (1). The etiological agents of the disease are spirochetes of the genus *Leptospira*, comprising over 250 pathogenic serovars (2). Certain serovars are known to be regionally endemic and reserved in certain species of wild mammals and domesticated animals. These carrier animals may remain asymptomatic but capable of transmitting leptospires to other animal species (incidental hosts) and humans, via direct contact with contaminated urine or indirectly through contaminated water and soil (3). Particularly, cattle have been reported to maintain serovars Hardjo, Sejroe and at times Pomona (3–5).

Despite evidence of both human and animal leptospirosis in Uganda, the epidemiology of the disease is still not well-investigated. Seropositivity to *Leptospira* species has been described in buffaloes (6) and in dogs (7), with the first case of clinical canine leptospirosis in Uganda reported recently (8). A random survey in beef and dairy cattle herds in two districts of Uganda revealed a seroprevalence of 19% (9). Additionally, Dreyfus et al. (10) demonstrated 35% prevalence of anti–*Leptospira* antibodies in health centre patients in Hoima, Uganda; with skinning of cattle during slaughter being significantly associated with the observed seropositivity. This further implicates cattle as potential sources of *Leptospira* infections to humans. Furthermore, renal carriage and/or shedding of pathogenic *Leptospira* was recently confirmed in 8.8% (*n* = 44) of slaughter cattle from the same population as this current study (11). The aim of this study was to determine the prevalence of anti-*Leptospira* antibodies by the standard microscopic agglutination test (MAT); and establish associated risk factors for *Leptospira* serostatus among slaughtered cattle. In order to assess the usefulness of serological tests as tools for surveillance of leptospirosis in cattle herds, we compared the performance of MAT against a *lipL32* based real time PCR (qPCR) assay conducted previously on the kidneys and urine of the same slaughter cattle tested in this study.

## Materials and Methods

### Study Design

We conducted a cross-sectional study between June and July 2017, in two purposively selected cattle abattoirs in Kampala, Central Uganda. The two abattoirs were Nsooba slaughter house, Kalerwe (AK) and City abattoir (LC). Selection of the two slaughter facilities was based on their large average daily slaughter volumes (162 cattle at AK and 221 at LC) and the broad regional diversity of source markets for their slaughter cattle. Cattle slaughtered at these abattoirs are gathered as individual animals (not herds) on a daily basis by various independent traders who buy them from independent farmers from geographically distinct locations of Uganda.

### Sample Size and Sampling Strategy

The sample size for estimating the seroprevalence with a 95% confidence level and precision of 0.05 was determined as 237 cattle, using Epitool calculators (12), and based on an estimated 19% prevalence earlier reported in a serological survey in dairy and beef herds in Uganda by Dreyfus et al. (9). Since we sampled individual animals and not herds, we did not have to take clustering into account. Blood samples were collected from a total of 500 randomly selected slaughter cattle, following the same sampling strategy that Alinaitwe et al. (11) used to co-currently collect matching kidney and urine samples from the same cattle population tested in this study (from the same animal, kidney, urine and blood/serum were collected; and serum tested separately by MAT).

In brief, the two abattoirs were visited on alternating week days for 21 days. At abattoir LC, four of the fourteen slaughter lines were randomly selected on each visit, and samples systematically collected. At the second abattoir (AK), there were no slaughter lines; and as such the facility was virtually divided into two spaces along its width. Animals slaughtered through one virtual space were opportunistically sampled, and the collections alternated between the two virtual spaces on subsequent visits. Here, random selection of individual animals from the pre-selected virtual space depended on the slaughter process itself. It would take 15–25 min to enroll and collect samples from a single animal. During this time another 5–8 animals would be laid down, which limited the probability that animals from the same population characteristics were selected.

### Sample Collection

At the time of evisceration, 4 ml of blood was collected from each randomly selected slaughter animal by cardiac puncture into a plain vacutainer (Becton Dickinson BD™). Additionally, animal demographic data was taken, and information on origin of the slaughtered cattle obtained from abattoir records or at times on consultation with the respective animal traders. The blood samples were kept on ice until delivery to the Central Diagnostic Laboratory at College of Veterinary Medicine, Animal Resources and Biosecurity (COVAB), Makerere University. At the laboratory, the blood was centrifuged at 2,000 g for 5 min, and serum harvested into cryogenic tubes for storage at −20°C.

### Serological Testing

The microscopic agglutination test (MAT) was used to determine presence of anti-*Leptospira* antibodies; in accordance with the OIE standards (13). A panel of 11 serovars (Table 1) representing 11 serogroups previously described as prevalent in Uganda (9) and those shown to be prevalent or maintained in cattle elsewhere in East Africa (14, 15) was employed. Briefly, seven day old live *Leptospira* cultures were used to screen the serum samples at an initial dilution of 1:50. Those with a positive reaction were then titrated in a serial 2-fold dilution to determine the end-point/titer (the reciprocal of the highest serum dilution at which ≥50% of the leptospires remained agglutinated). Sera with a titer ≥100 against any *Leptospira* serovar were considered positive. Sera samples that reacted to one or more *Leptospira* serovars were considered as positive for all the reacting serovars, despite any disparities between antibody titers detected against each of the reacting serovars.

**Table 1:** Strains of *Leptospira* species used as live antigens in the MAT.

**Genomospecies**	**Serogroup**	**Serovar**	**Strain**
*L.interrogans*	Australis	Australis	Ballico
	Icterohemorrhagiae	Icterohemorrhagiae	RGA
	Pomona	Pomona	Pomona
	Canicola	Canicola	Strain Hond Utrecht IV
	Djasiman	Djasiman	Djasiman
*L. kirschneri*	Autumnalis	Butembo	Butembo
	Grippotyphosa	Grippotyphosa	Duyster
*L. borgpetersenii*	Pyrogenes	Nigeria	Vom
	Tarassovi	Tarassovi	Perepelitsin
	Sejroe	Sejroe	M84
*L. santarosai*	Shermani	Shermani	1342K

### Real-Time PCR Assay

Data on renal *Leptospira* infection (shedding and/ or carriage of pathogenic *Leptospira* species) was provided by Alinaitwe et al. (11), who had already conducted a Taqman real-time PCR (qPCR) assay on kidney homogenates and urine of the same slaughter cattle whose matching sera samples we have tested by MAT in this study. The qPCR targeted the gene *lipL32* which encodes for a major outer membrane protein, only present in pathogenic *Leptospira* species (16).

### Data Analysis

We recorded the data in Microsoft Excel 2010 (Microsoft Corp, Redmond WA, USA) and analyzed it in Stata 15 (Stata Corp., USA). The overall prevalence of seropositive animals and seroprevalence by serogroup (serovar) were calculated. The association between seroprevalence and the exposure variables “source abattoir,” “age,” “sex,” “breed,” and “region of origin” of slaughtered cattle were analyzed by univariable logistic regression. Further, in a manual forward selection method, we assessed the association between these exposure variables and *Leptospira* seropositivity by multivariable logistic regression analysis. Exposure variables were each entered in the model and were kept in the model if the likelihood ratio test was statistically significant (*p* ≤ 0.05) compared to the nested model. The performance of the MAT against the qPCR (the latter being the reference) was assessed based on the test's sensitivity, specificity, predictive values and level of agreement as determined by the Cohen Kappa statistic (17). Since we tested individual animals from the slaughterhouse and not herds, we did not account for clustering.

### Ethical Considerations

The study procedures were approved by the Uganda National Council for Science and Technology (A565), and consent from abattoir representatives was obtained ahead of the study and also at the time of sampling.

## Results

### Study Population Characteristics

Of the 500 slaughter cattle sampled, 468 (93.6%) were adult cattle (≥1.5 years), and there were slightly more cows (54.4%) than bulls. The majority of the cattle slaughtered at the abattoirs were sourced from the central (40.0%) and western (13.8%) regions of Uganda. Up to 8.4% of the slaughter cattle were reportedly sourced across the borders of Uganda (mainly Tanzania), while the definite origin of up to 19.0% of the cattle could not be established due to insufficient accompanying documentation from their source markets. Indigenous breeds of cattle dominated the slaughter population at the two study abattoirs (80.2%) as compared to their exotic and cross-bred counterparts (Table 2).

**Table 2:** Population characteristics of the sampled slaughter cattle (*N* = 500), *Leptospira* seroprevalence and associated risk factors as calculated by univariable logistic regression.

**Variable**	***n* (%)**	**Prev %**	**OR**	**95% CI**	***P*-value**
**Breed**					
Local	401 (80.2)	26.4	Ref		
Cross	94 (18.8)	31.9	1.3	0.8–2.1	0.285
Exotic	5 (1.0)	60.0	4.2	0.7–25.3	0.12
**Sex**					
Female	272 (54.4)	29.4	Ref		
Male	228 (45.6)	25.9	0.8	0.6–1.2	0.38
**Age**					
Juvenile (<1.5 Years)	32 (6.4)	12.5	Ref		
Adult	468 (93.6)	28.9	2.8	1.0–8.2	0.055
**Region**					
Central	200 (40.0)	28.5	Ref		
Western	69 (13.8)	33.3	1.3	0.7–2.3	0.45
Northern	39 (7.8)	25.6	0.9	0.4–1.9	0.72
Eastern	55 (11.0)	21.8	0.7	0.3–1.4	0.33
Across Boarder	42 (8.4)	26.2	0.9	0.4–1.9	0.76
Undetermined	95 (19.0)	27.4	1.0	0.6–1.6	0.84
**Abattoir**					
AK	187 (37.4)	26.2	Ref		
LC	313 (62.6)	28.8	1.1	0.8–1.7	0.538

*n is the number of observations under each variable level, and Prev% is the percentage Leptospira seroprevalence by variable level (population characteristic). Ref is the parameter in a given category to which the other parameters in the same category were compared*.

*Local collectively refers to breeds of cattle indigenous to Uganda while exotic to those originating outside Uganda. Cross refers to an offspring of interbreeding between a local and exotic breed*.

*MAT, microscopic agglutination test; OR, odds ratio and CI, confidence interval*.

### Prevalence of Anti-Leptospira Antibodies

Of 500 cattle tested, 27.8% (95% CI 23.9–32.0) (*n* = 139) tested positive (titer ≥ 100) to at least one *Leptospira* serovar. The majority of seropositive cattle reacted to serovars Tarassovi (sg Tarassovi) (11.6%), Sejroe (Sg Sejroe) (7.8%), and Australis (Sg Australis) (5.2%); with no cattle reacting to serovar Djasman (Table 3). Seropositivity to multiple *Leptospira* serovars was detected in 4.4% (22/500) of cattle, and up to 15.8% (22/139) of the seropositive cattle had high anti-*Leptospira* antibody titers (≥800).

**Table 3:** Prevalence and levels (titers) of serovar-specific anti-*Leptospira* antibodies measured by the microscopic agglutination test (MAT) among cattle slaughtered at major Ugandan abattoirs (*N* = 500).

	**MAT titer**							
**Serovar**	**100**	**200**	**400**	**800**	**1,600**	NPos^*^ serovar	**% Positive**	**95% CI**
Tarassovi	10	12	21	13	2	58	11.6	8.9–14.8
Sejroe	23	13	3	0	0	39	7.8	5.7–10.6
Australis	19	6	0	0	1	26	5.2	3.5–7.6
Shermani	0	6	5	2	0	13	2.6	1.4–4.5
Grippotyphosa	4	1	1	0	0	6	1.2	0.5–2.7
Pomona	0	3	0	1	1	5	1.0	0.4–2.4
Icterohemorrhagiae	1	2	1	1	0	5	1.0	0.4–2.4
Canicola	0	2	1	1	0	4	0.8	0.2–2.2
Nigeria	1	1	1	0	0	3	0.6	0.1–1.9
Butembo	2	0	0	0	0	2	0.4	0.07–1.6
Djasman	0	0	0	0	0	0	0.0	0.09–0.9
Npos^*^ titer	60	46	33	18	4	161		

Npos^*^titer refers to the number of cattle with the various levels of antibodies (titers) and Npos^*^ serovar to those reacting against the respective Leptospira serovars/serogroups tested.

*CI, Confidence interval; MAT, microscopic agglutination test*.

### Risk Factors for Seroprevalence of Pathogenic *Leptospira* Species

In the univariable logistic regression model, none of the exposure variables were significantly associated with cattle having antibodies against leptospires at a *p* ≤ 0.05 (Table 2). Nevertheless, older animals had 2.8 times (95% CI 0.98–8.24) greater odds of being seropositive than younger ones (<1.5 years), albeit with a *p*-value of 0.055. None of the exposure variables improved the model fit in the multivariable logistic regression model (data not shown).

### Performance of the MAT Against the lipL32 qPCR Assay

Of the 44 qPCR positive samples reported by Alinaitwe et al. (11), matching sera from 23 were found to be positive by MAT as well. Overall there was a fair agreement between the MAT and the qPCR results (Cohen's kappa statistic 0.21; *P* < 0.001). The sensitivity and specificity of the MAT over the qPCR were 65.9% (95% CI 50.1–79.5) and 75.9% (95% CI 71.7–79.7), while the positive predictive and negative predictive values were 20.9 and 95.8%, respectively. Furthermore, the performance of MAT against the qPCR assay was explored at different MAT cutoffs. While the sensitivity and NPV decreased with increasing MAT titer cut-off, the specificity and PPV increased (Table 4).

**Table 4:** Performance of the MAT against the *lipL32* qPCR assay at various titer cut-offs.

**MAT titer cut-off**					
**Test parameter**	**100**	**200**	**400**	**800**	**1,600**
Sensitivity (CI)	65.9 (50.1–79.5)	52.3 (36.7– 67.5)	31.8 (18.6–47.6)	18.2 (8.2–32.7)	4.6 (0.6–15.5)
Specificity (CI)	75.9 (71.7–79.7)	84.2 (80.5–87.4)	91.5 (88.5–93.9)	97.2 (95.2–98.5)	99.6 (98.4–100.0)
PPV (CI)	20.9 (16.8–25.6)	24.2 (18.3–31.3)	26.4 (17.5–37.8)	38.1 (21.3–58.4)	50.0 (12.6–87.4)
NPV (CI)	95.8 (93.8–97.2)	94.8 (93.1–96.2)	93.3 (91.9–94.5)	92.5 (91.5–93.4)	91.5 (91.0–92.0)

*PPV, positive predictive value; NPV, negative predictive value; CI, confidence interval*.

## Discussion

Detection of a high prevalence of anti-*Leptospira* antibodies against several *Leptospira* serogroups/serovars in cattle slaughtered at Ugandan abattoirs indicates that cattle are exposed to various *Leptospira* serogroups and serovars. This result supplements already existing data from studies that previously demonstrated anti-*Leptospira* antibodies in cattle (6, 9), and one that confirmed renal *Leptospira* infection in cattle from various areas of Uganda (11). The latter study was conducted in the same abattoirs as the current study. The studied abattoirs are currently the largest in Uganda (in terms of daily slaughter volume), and source their slaughter animals from a wide geographical range, thus reducing sampling bias. Additionally, cattle slaughtered at these abattoirs were never contributed by selected herds but rather these were individual animals independently sold to various cattle traders daily and by independent farmers from geographically distinct locations of Uganda. Seropositive animals were detected among cattle sourced from all the five regions, with no significant association between seropositivity and any of the regions of origin. Therefore, we postulate that leptospirosis is endemic and widely spread in several cattle populations in Uganda. A similar level of *Leptospira* prevalence as found in this study has been reported in cattle in Tanzania (14, 18), and in Kenya (19), with serovars Tarassovi and Hardjo (Hardjo is in the same serogroup as Sejroe used in the current study) being more prevalent. Tanzania and Kenya are neighbors with Uganda and the three countries share a similar ecology. This may mean that similar factors influence the leptospirosis burden in the three countries. In addition, there is evidence of trans-boundary movement of animals including cattle between these countries (20, 21). Up to 15.8% of the seropositive cattle had high anti-*Leptospira* antibody titers (≥800), probably indicating they had recently been infected with the respective *Leptospira* species at the time of sampling. The association between seropositivity and age of cattle as observed in this study could be explained by the higher likelihood of exposure to *Leptospira* contaminated sources with increasing age, especially in endemic settings.

Previous studies in Uganda have so far demonstrated circulation of the cattle maintained serovar Hardjo (6, 11). In the current study, we found high reactivity to Sejroe, a serovar in the same serogroup as Hardjo. Cross-reactivity between serovars of the same serogroup has been demonstrated before (15, 22). However, the prevalence of Hardjo in Ugandan cattle seems lower than reported elsewhere in East Africa; probably indicating the role of other serovars in *Leptospira* infection in Ugandan cattle. The low seroprevalence of rodent associated serogroups/serovars, such as Icterohemorrhagiae and Grippotyphosa in this study is in agreement with previous studies conducted on cattle (9) and in humans (10) in Uganda. This raises questions on the role of rodents in maintenance and transmission of *Leptospira* species in Uganda. It remains unclear whether rodents carry different serovars from those used on the test panels in Uganda or if the level of environmental contamination by rodents is generally low to permit an indirect transmission. In this regard, we already are testing kidneys collected from rodents trapped from several ecological sites, to establish the renal carriage of pathogenic *Leptospira* and genomic identity of *Leptospira* species carried by rodents in Uganda. In the present study, we found a high prevalence of anti-*Leptospira* antibodies against serovar Tarassovi, which has also been reported in cattle (18, 23) and pigs (24, 25) in East Africa and elsewhere. Though there are not many published reports of leptospirosis in other species of animals in Uganda, we still think that the nature of animal husbandry practices may facilitate interspecies interactions and spread of *Leptospira* infection. In rural Uganda (where the majority of slaughter cattle were sourced), small scale farmers may leave their cattle to free-range with several other animals including goats, sheep, dogs, and swine, hence increasing interspecies interactions. Additionally, the free-ranging herds often drink from common open water sources that may play an important role in indirect transmission of leptospirosis. Australis, a serovar found prevalent in cattle in the current study was diagnosed in 6.1% (*n* = 23) of the 379 pigs tested in a survey conducted on rural piggery farms in South Western Uganda (unpublished data). Such interspecies interactions not only make the transmission cycle of leptospirosis more complex but may also magnify the disease burden in livestock, especially if infection occurs with non-adapted serovars. While leptospirosis may be widespread among cattle herds in Uganda, the infecting *Leptospira* serovars seem to vary regionally. For example, in two districts representing the Northern and Eastern regions of Uganda, Pomona seroprevalence was highest with 9.5% (6.4–13.7%), followed by Kenya 5.1% (2.9–8.6), Nigeria 4.0% (2.1–7.2), Wolfii 3.3% (1.6–6.3), Butembo 1.9% (0.7–4.4), and lastly Hardjo 1.5% (0.5–3.9) (9); yet Tarassovi, Sejroe (same serogroup as Hardjo) and Australis were the most prevalent in the current study. This may imply that seasonal and geographical differences have influence on the epidemiology of leptospirosis, and thus the need to institute *Leptospira* surveillance programs at a regional level.

At a herd level, direct non-invasive molecular detection of *Leptospira species* in urine could be the best approach to assess *Leptospira* infection status and associated risk of transmission to other species (including humans). However, serological testing, including the MAT is currently still more widely available and one of the most common diagnostic tools used to collect surveillance data on leptospirosis in both humans and animals. The MAT indirectly measures *Leptospira* exposure and/or infection through detection of specific antibodies against *Leptospira* serogroups/serovars. In the current study, we attempted to compare MAT output with output of a qPCR assay and only found a fair agreement of 0.21 (Cohen's kappa statistic *P* < 0.001) between the two tests. This may be expected since: 1. in carrier animals *Leptospira* infection may not induce long lasting natural immunity, yet leptospires persist in the kidneys of these animals for several months or years. 2. In an endemic setting, animals may get recurrent infections through exposures to contaminated environments (become seropositive), but only intermittently shed leptospires in urine, limiting chances of detection by molecular techniques. In addition, detectable levels of *Leptospira* antibodies may persist in cattle that have recently been treated with certain antibiotics that reduce urinary shedding. MAT as used in this study was found to be highly specific, and had a high negative predictive value when compared to the qPCR assay, implying a rather strong association between sero-negativity and absence of renal *Leptospira* infection. However, the reliability of MAT predictability for renal *Leptospira* infection should be taken with a lot of caution since predictive values of diagnostic tests depend on prevalence (assuming similar sensitivity and specificity). Therefore, the reportedly high negative predictive value of MAT in this study may change significantly under other prevalence scenarios. Since there is already evidence of good performance of ELISA (26, 27) and point-of-care diagnostics (28, 29) for human leptospirosis, it may be necessary to validate these and more such serological assays for veterinary use. These would then serve as cheaper screening options for use in animal leptospirosis surveillance programs in low resource settings, including Uganda. Nevertheless, for confirmation of clinical cases of leptospirosis, a qPCR result of urine or MAT on paired sera is recommended.

## Conclusions

Findings of anti-*Leptospira* antibodies among slaughter cattle in this study implies exposure of cattle to leptospires; with older cattle (≥1.5 years) having higher odds of being exposed than younger ones. Cattle in Uganda are commonly exposed to serovars Tarassovi (Sg Tarassovi), Sejroe (Sg Sejroe), and Australis (Sg Australis), with potential to expose humans and other species by shedding *Leptospira* species at the human-livestock-environment interface. Individuals in close contact with cattle, including abattoir workers, those involved in obstetrics, milking, and animal transportation may be at highest risk. This risk and the general leptospirosis burden should be assessed in the human population in Uganda. And if leptospirosis is shown to be a health problem in humans (what the authors strongly hypothesize), vaccination of cattle herds together with treatment of infected animals, and protection of water sources could be some of the control strategies at the animal level (farms). Other indirect measures may include sensitization of workers in risky occupations and use of appropriate personal protective equipment.

The high specificity and negative predictive value of MAT as used in this study when compared to the qPCR assay may imply a rather strong association between seronegativity and absence of renal *Leptospira* infection. However, MAT predictability for renal *Leptospira* infection may be interpreted cautiously since predictive values of diagnostic tests are dependent on prevalence.

## Data Availability Statement

The datasets generated for this study are available on request to the corresponding author.

## Ethics Statement

The study procedures were approved by both the institutional review board of the College of Veterinary Medicine, Animal Resources and Biosecurity (COVAB), Makerere University (SBLS/REC/17/003) and the Uganda National Council for Science and Technology (A565). We also obtained consent of abattoir representatives ahead of the study and also at the time of sampling.

## Author Contributions

LA and AD conceived the study. LA, DN, and PP participated in the collection of data, while LA and AD conducted the statistical analyses and AD and CK oversaw the study. LA coordinated the drafting of the article and AD was the main reviewer.

### Conflict of Interest

The authors declare that the research was conducted in the absence of any commercial or financial relationships that could be construed as a potential conflict of interest.
